# The Relationship between Body Mass Index and Depression among High School Girls in Ahvaz

**DOI:** 10.1155/2016/3645493

**Published:** 2016-09-01

**Authors:** Ashraf Tashakori, Forough Riahi, Amin Mohammadpour

**Affiliations:** Department of Psychiatry, Golestan Hospital, Ahvaz Jundishapur University of Medical Sciences, Ahvaz 6135733118, Iran

## Abstract

*Objective*. Today, obesity and depression are two major illnesses that are on the rise all over the world and threaten human health. This research was done to determine the relationship between Body Mass Index (BMI) and depression among Ahvaz high school female students.* Method*. In a descriptive-analytical study using stratified random sampling, 400 female high school students in academic year of 2013-2014 were picked and their height and weight were measured. BMI was classified based on World Health Organization classification. To assess the severity of depression, Beck depression questionnaire was used. In order to analyze the data, descriptive statistics and Pearson correlation test were used.* Results*. In terms of BMI 9% of students were slim, 77% were at an acceptable level, and 14% were overweight. Also, the prevalence of depression was 86.20% major depression and 13.79% moderate depression for obese persons, 10.41% major depression and 70.83% moderate depression for overweight persons, 8.78% major depression and 12.97% moderate depression for normal weight persons, and 9% moderate depression for slim persons. The relationship between BMI and depression among high school students is positive and significant (*P* < 0.001; *r* = 0.555).* Conclusion*. There is a positive and significant relationship between BMI and the severity of depression among Ahvaz high school female students.

## 1. Introduction

A rising public health problem around the world is obesity, and depression is one of the most common mental disorders [1]. Experts suggest that increasing incidence of depression is related to the high prevalence of obesity [2]. The pervasiveness of overweight and obesity has constantly increased among all community groups in the past 2-3 decades in America and if this trend goes on by 2030 about 86.3% of adults will be obese or overweight [3]. Obesity is a disease of indolence and results from a prototype of inactivity which is attributive of the automated life [4]. On the other hand depression is a mood disorder that affects the mental health. Given the high prevalence of depression and the significant burden of the disease on the individual, health system, and society, adopting appropriate methods to identify risk factors, prevention, treatment, and management of this illness is a must [1, 5–7]. Affecting the mental health in turn disturbs individual's social and physical health. Depression leads to disruption in job performance and social and interpersonal relations [1]. Depression among high school students is an important issue because it lowers their academic success and achievements [8].

The effects of obesity on physical health have been well documented, but its consequences for mental health are less certain. There are several studies relating to the association of obesity and depression in which some approve [9–15] and others reject [16, 17] the relationship. There are generally three types of studies relating depression and obesity. The first type is the ones that recognize role of obesity in depression and suggest that self-body image is effective in depression [16, 17]. The second type is the ones that blame depression in obesity [18–21]. The third type is the ones that only try to determine the relationship between the two variables [14, 15]. Age and gender have associations with depression and obesity. Onset of depression in adolescence doubles the danger of obesity in comparison with those who do not suffer depression. Adolescent depression due to obesity is more seen in women. Some studies even report that existence of depression in adolescence increases the individual's potential for developing obesity in older age [19].

Iran like many developing countries is experiencing the global obesity epidemic and the resulting adversaries [22] and the prevalence of depression among Iranian populations ranged from 5.69 to 73 percent [5]. However, a few studies have been found that surveyed the relationship between obesity or Body Mass Index (BMI) and depression among adults in Iran.

What necessitates the current research is the lack of enough information on the relation between obesity and depression in various age groups and as a result the lack of such information in Ahvaz city and limited research in women's field particularly among adolescents.

## 2. Material and Methods

This descriptive-analytical study was approved by Research Board Committee of Ahvaz University of Medical Sciences. The population of this research was all female high school students of Ahvaz city who were in the academic year of 2013-2014. To determine the sample size, Kerjesi and Morgan model was used [23] and the sample size of 400 was obtained. Sample units were selected using two-stage cluster sampling and the proportions of students in various places of the city in the sample size were consistent with their proportion in the statistical population size. Ahvaz city consists of four districts of education and training and we did our survey after obtaining permission from the state educational office and its four-district branches. Inclusion criteria were second and third grade students of Ahvaz high schools who had not experienced divorce, death, or remarriage of their parents and who did not have any acute or chronic physical and psychiatric diseases. This information was obtained through students' medical records and report of students.

*BMI Acquisition*. After making an appointment with the high school principal and getting consent for participation and determining a preset time, each day we visited schools on time and after gaining their trust the questionnaire was distributed and after receiving it back, their height and weight were measured. BMI is the best way to measure overweight and obesity. Overweight in adults is defined as BMI over 25 kg/m^2^ and BMI over 30 kg/m^2^ is defined as obesity and BMI less than 18.5 is distinguished as being slim [24]. In order to measure height and weight of the participants, physical tools with medium ratio were used. Height and weight were measured by an authority figure in the school. Weight of the participants was measured with light clothing and without shoes with a digital scale with accuracy of 0.1 kg. Its measurement and accuracy were checked during various stages using standard weights. Height of the participants was also checked using a measurement tape with accuracy of 0.1 cm which was fixed to the wall with a special tool. The participants took off their shoes and heels; buttocks, shoulders, and back of the head touched the wall, and the Frankfort line was parallel to the ground.

*Assessment of Depression*. In order to assess depression Beck Depression Inventory (BDI) was used. The BDI is one of the most widely used instruments not only for assessing the intensity of depression in patients with depressive disorders, but also for screening of depression in normal populations [25]. It has acceptable psychometric properties in Iranian population [26]. The questionnaire consisted of 21 questions with 4 answers and the participants could fill it in within minutes. The participants were asked to read every question carefully, and among the options they were asked to tick the one that best describes their past and present condition. The answers to each question had a score from zero to three. The sum of this test's scores fluctuated between 0 and 63 scores. Scores 0–9 denoted normality, scores 10–16 marked mild depression, scores 17 to 29 marked moderate depression, and scores 30–63 indicated major depression [25].

We used descriptive statistics method in order to categorize information, display the categorized information in frequency tables, formulate the proportion of frequency, and draw graphs. Also, we employed Pearson correlation test with the help of statistical software SPSS20 for determining the relationship between BMI and depression among students.

## 3. Results

In this study 400 female students were evaluated. A total of 400 potential participants actually participated. Fifty students filled questionnaire incompletely; thus, we asked them to fill questionnaire again. The mean of height and weight of these students was 161.1 ± 5.9 cm and 57.6 ± 14.1 kg, respectively. The pervasiveness of Body Mass Index based on the World Health Organization standard showed that from all the participants 29 (7.3%) were obese, 48 (12%) were overweight, 239 (59.8%) were at an acceptable level, and 84 (21%) were slim. The comparison of the frequency of depression in the four distinctive BMI groups showed that all obese persons in this study (29) suffered severe and moderate degrees of depression and the majority of them (86.20%) suffered major depression. In persons with overweight only 8.33% were normal and the rest of the participants suffered various degrees of depression. The most common degree in this group of depression was 70.83% moderate depression. In persons with normal BMI, 77% suffered depression and the most common type of depression was minor depression. In the slim group the majority was healthy (59.52%) and in this group major depression was not reported. And moderate depression in this group was 10.71% and minor depression was 29.76% (Table 1).

**Figure 1:**
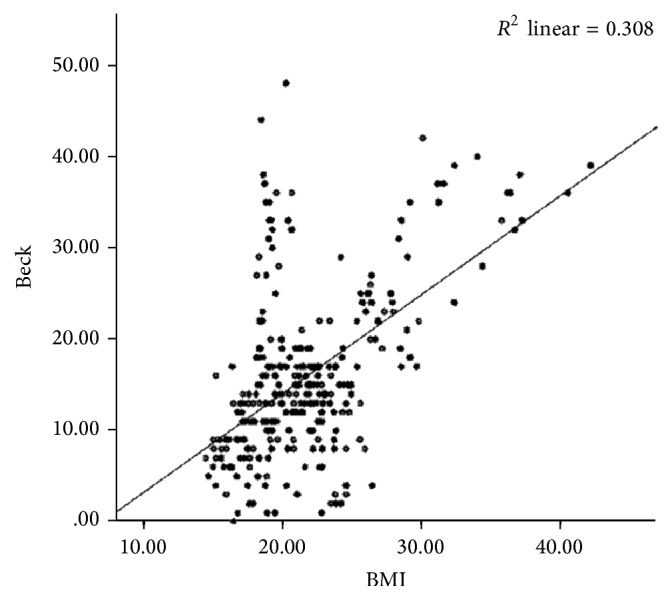
The relationship between BMI of high school female students and their degree of depression.

For assessing the relationship between female high school students' BMI and the degree of depression Pearson correlation coefficient test was used and a positive and significant relationship was observed between BMI and the degree of depression (*P* < 0.001) (*r* = 0.555) (Figure 1).

## 4. Discussion

The results showed that there is a positive and significant relationship between BMI and depression in high school female students. In other words, there is a significant relationship between obesity and the degree of depression in female adolescents. There are some explanations for this relation. Markowitz et al. conducted a review of the literature for understanding the relation between obesity and depression. In this review cross-sectional studies, longitudinal studies, and the evidence based on intervention studies suggest that obesity is associated with depressive symptoms. The longitudinal studies suggest that obesity can lead to later depression and intervention studies suggest that weight loss treatment improves mood, but this improvement may not be result of actual weight loss. Authors of this review believe that contribution of obesity to later depression is a complex relation and propose two causal paths based on the moderators of the relation between obesity and depression: “health concern” pathway and “appearance concern” pathway. In “health concern” pathway depression in severely obese individuals is the result of functional impairment and poor self-rated health. In “appearance concern” pathway, severely obese women and high socioeconomic individuals may be more predisposed to depression through body image dissatisfaction, repeated dieting, and stigma. They propose several mechanisms including self-rated health, behavioral or cognitive factors, and physiological or immunological factors in bidirectional relation between obesity and depression. Finally, their proposed mechanisms of causal pathway from depression to obesity are behaviors such as poor exercise, cognition such as negative thoughts, and social mechanisms such as reduced support [27]. Other explanations for the relation between obesity and depression in women may be a shared genetic risk for both conditions [13].

Our results are consistent with those of other studies [2, 9–12, 14, 15, 19, 27] and suggest that there is a significant relationship between obesity and the degree of depression in female adolescents.

de Wit et al. conducted a meta-analysis of community-based studies in which the association between depression and obesity was examined in adults and showed a significant positive association between depression and obesity in the general population, especially among women [9]. Keddie findings showed an association between obesity and depression in severely obese women. But, adjusting for number of chronic conditions, self-rated health status and demographic variables weakened this association [10]. Luppino et al. in a systematic review and meta-analysis of longitudinal studies demonstrated a reciprocal association between depression and obesity. They found that baseline obesity can increase following depression and this association is more robust for depressive disorder than for depressive symptoms and for Americans than for Europeans [12]. Dong et al. suggested that extreme obesity is related to the increased risk for depression across gender and racial groups, even after controlling for chronic physical disease, familial depression, and demographic risk factors [11]. Arterburn et al. suggested strong relationships between depression, obesity, and disability [14].

Our results differ from those of Askari et al. that showed that obesity does not lead to depression [16]. This inconsistency may be due to different sampling. In their study male and female adult individuals in Yazd health centers were chosen but in our study participants were female students in high school. Also, the findings of the current study seem to be inconsistent with those of Roberts et al.'s study that found no independent association between major depression and body weight in adolescents from the community. They suggested components of body image as an etiologic link between major depression and body weight among adolescents [17].

The association between obesity and depression may be affected by sociodemographic factors or other conditions. These potential moderator or mediator factors may lead to the conflicting results in the association between obesity and depression [27, 28]. Moderator factors, such as severity of depression, severity of obesity, gender, socioeconomic status, gene-environment interactions, and childhood experiences, specify individuals and conditions where effects of agents occur. Mediator factors, such as eating, physical activity, teasing, eating problems, and stress, identify mechanisms of the causal pathway between agents [28].

One limitation of our study was the reliance on self-report questionnaire for determination of depressive symptoms and we did not assess categories of depressive disorders diagnostically. The strength of our study was large sample size and measurement of height, weight, and BMI through standard methods by researchers team.

In summary, the results showed that there is a positive and significant relationship between BMI and depression in high school female students in Ahvaz city and these conditions should be routinely screened and treated in them. The relationship between BMI and depression may be different across different ethnic or cultural groups; thus, we suggest such studies among other groups.

## Figures and Tables

**Table 1:** Prevalence distribution of female high school students according to BMI and the degree of depression (based on Beck depression questionnaire).

BMI	Depression									
	Healthy		Minor depression		Moderate depression		Major depression		Total	
	*f* ^*∗*^	*P* ^*∗∗*^ (%)	*f*	*P* (%)	*f*	*P* (%)	*f*	*P* (%)	*f*	*P* (%)
Underweight	50	59.52	25	29.76	9	10.71	0	0	84	100
Normal weight	55	23.01	132	55.23	31	12.97	21	8.78	239	100
Overweight	4	8.33	5	10.41	34	70.83	5	10.41	48	100
Obese	0	0	0	0	4	13.79	25	86.20	29	100

^*∗*^Frequency.

^*∗∗*^Prevalence.
